# Optimal Diagnostic and Treatment Practices for Facial Dysostosis Syndromes: A Clinical Consensus Statement Among European Experts

**DOI:** 10.1097/SCS.0000000000010280

**Published:** 2024-05-27

**Authors:** Victor L. Van Roey, Willemijn F.E. Irvine

**Affiliations:** * Department of Plastic and Reconstructive Surgery, Erasmus MC, University Medical Center Rotterdam, Rotterdam, The Netherlands; † European Reference Network for Rare and/or Complex Craniofacial Anomalies and Ear, Nose, and Throat Disorders, Rotterdam, The Netherlands; ‡ Department of Pediatric Surgery, Erasmus MC, University Medical Center Rotterdam, Rotterdam, The Netherlands; § Department of Evidence-Based Medicine and Methodology, Qualicura Healthcare Support Agency, Breda, The Netherlands

**Keywords:** Delphi technique, Acrofacial dysostosis, Mandibulofacial dysostosis, Treacher Collins syndrome, Nager syndrome, Genee-Wiedemann syndrome

## Abstract

Facial dysostosis syndromes (FDS) are rare congenital conditions impacting facial development, often leading to diverse craniofacial abnormalities. This study addresses the scarcity of evidence on these syndromes about optimal diagnostic and treatment practices. To overcome this scarcity, European experts from ERN CRANIO collaborated to develop a clinical consensus statement through the Delphi consensus method. A systematic search of Embase, MEDLINE/PubMed, Cochrane, and Web of Science databases was conducted until February 2023. The quality of evidence was evaluated using various tools depending on the study design. Statements were subsequently formed based on literature and expert opinion, followed by a Delphi process with expert health care providers and patient representatives. In total, 92 experts from various specialties and three patient representatives were involved in the Delphi process. Over 3 voting rounds, consensus was achieved on 92 (46.9%), 58 (59.2%), and 19 (70.4%) statements, respectively. These statements cover the topics of general care; craniofacial reconstruction; the eyes and lacrimal system; upper airway management; genetics; hearing; speech; growth, feeding, and swallowing; dental treatment and orthodontics; extracranial anomalies; and psychology and cognition. The current clinical consensus statement provides valuable insights into optimal diagnostic and treatment practices and identifies key research opportunities for FDS. This consensus statement represents a significant advancement in FDS care, underlining the commitment of health care professionals to improve the understanding and management of these rare syndromes in Europe.

Facial dysostosis syndromes (FDS) are rare congenital conditions that mainly affect the development of the bones and soft tissues of the face. These syndromes can cause a range of craniofacial abnormalities, including eyelid abnormalities (ie, eyelid coloboma), ear abnormalities (ie, microtia or cup-ear deformities), underdeveloped cheekbones (ie, malar hypoplasia), a small maxilla and mandible (ie, maxillomandibular hypoplasia), and a cleft lip and/or palate, which can result in functional problems.^1–5^ Some of these syndromes, such as Nager and Miller syndrome, also affect limb development.^6^ Altogether, these conditions can significantly impact a patient’s quality of life,^7,8^ with many requiring multiple complex medical interventions and ongoing support related to breathing, feeding, speech, and hearing, as well as psychological and sociological well-being, oral health, ocular problems, and reproduction.

Currently, there is limited scientific evidence on the optimal diagnostic and treatment practices for FDS. This is mostly due to their rarity, with the most common syndrome (ie, Treacher Collins syndrome) having a prevalence of 1 to 9 per 100,000 live births,^9^ and the exact prevalence of some FDS (eg, Miller and Nager syndrome) still being unknown.^10,11^ Besides, there is great variability in the severity of symptoms, and the multidisciplinary treatment is often personalized, complex, and lengthy. Thus far, this has hindered the development of a reliable clinical practice guideline for FDS.

Nevertheless, to address the challenges of diagnosing and treating FDS, and uncover research opportunities, a group of European experts has come together to develop a clinical consensus statement. This group consisted of all the European expert centers for FDS that are members of the ERN CRANIO (European Reference Network for rare and/or complex craniofacial anomalies and ear, nose, and throat disorders; https://www.ern-cranio.eu/). ERN CRANIO is a network of European health care providers coordinated from Rotterdam, the Netherlands. ERN CRANIO aims to pool together disease-specific expertise, knowledge, and resources from across Europe to achieve health goals that may otherwise be unachievable in a single country.

Overall, the goal of this clinical consensus statement is to provide statements reflecting expert consensus on optimal diagnostic and treatment practices and needed research for FDS through the Delphi consensus method. The outcomes of this study will serve to inform health care providers, FDS patients, and their parents, and contribute to progress toward optimal care for all FDS patients in Europe. The key findings of this consensus statement are presented, including statements on general care; craniofacial reconstruction; eyes and lacrimal system; upper airway; genetics; speech, hearing; dental treatment and orthodontics; growth, feeding, and swallowing; extracranial anomalies; and psychology and cognition. It should be noted that this article exclusively refers to Treacher Collins (ie, Franceschetti-Klein syndrome, mandibulofacial dysostosis), Nager (ie, preaxial acrodysostosis, mandibulofacial dysostosis with preaxial limb anomalies, NAFD), and Miller (ie, Genée-Wiedemann syndrome, postaxial acrodysostosis, mandibulofacial dysostosis with postaxial limb anomalies, POADS) syndromes among the various forms of FDS.

## METHODS

The Clinical Consensus Statement Development Manual was used as a guide for the current statement,^12^ as well as the recommendations for the Conducting and REporting of DElphi Studies (CREDES).^13^ The entire process could be divided into 2 phases, starting with the preparation phase, made up of a systematic literature search, quality appraisal, and formation of statements. The subsequent voting phase involved Delphi surveys to identify areas of agreement and disagreement as to what are optimal diagnostic and treatment practices and needed research for FDS. Throughout the process, a panel of experts was consulted for substantive matters. The expert panel was composed of health care providers from each specialty directly involved in the care of FDS patients, all from FDS expert centers within the ERN CRANIO (Supplemental Digital Table 1, Supplemental Digital Content 1, http://links.lww.com/SCS/G243). In addition, the panel included 3 patient representatives affiliated to national patient organizations for rare (congenital craniofacial) conditions (ie, Laposa and Rare Diseases Croatia).

### Literature Search

The Embase, MEDLINE/PubMed, Cochrane, and Web of Science databases were systematically searched for English articles addressing FDS, published from January 1985 to February 2023 (Search strings in Supplemental Digital File 1, Supplemental Digital Content 2, http://links.lww.com/SCS/G244). The title and abstract of all obtained articles were independently screened by 3 reviewers for correspondence with our eligibility criteria. The full texts of the remaining articles were, subsequently, screened using the same criteria. Differences in the included articles between reviewers were resolved through discussion. Rayyan Systematic Review web tool was used for the entire article selection process.

All original studies reporting on the treatment or diagnostics of FDS or their sequelae, including at least 5 human FDS patients, were eligible for inclusion. Studies including patients with other syndromes were also eligible if the results of FDS patients were reported separately. Studies exclusively investigating the etiology, pathogenesis, or preoperative morphology of FDS were excluded, as well as conference proceedings, commentaries, letters, narrative reviews, editorials, dissertations, and unpublished evidence. In case of evidence gaps, panel members were allowed to supplement more general articles on the topic, including patients with other craniofacial anomalies, if considered appropriate.

### Quality Appraisal

All included studies were classified according to the level of evidence of the Oxford Centre for Evidence-Based Medicine (2009).^14^ The quality of the included studies was additionally assessed using a variety of tools depending on the study design, including the FLC 3.0 web application^15^ for case series, cohort studies, and case-control studies, the tool for critical evaluation of cross-sectional studies,^16^ the Joanna Briggs Institute Critical Appraisal tool^17^ for quasiexperimental studies, and the CASP Checklist^18^ for qualitative studies. Quality appraisal was independently performed by 2 blinded reviewers. Accordingly, the quality of each included study was categorized as high, average, or low.

### Statement Formation

Statements were derived from the discussion and conclusion sections of the included articles. The list of statements was, subsequently, complemented with statements based on expert opinion by members of the expert panel, especially for topics where evidence was scarce or lacking. To identify these evidence gaps, topics of interest were listed by the expert panel before the literature search. Finally, opinions from patient representatives outside of the panel were obtained through a patient survey (Supplemental Digital File 2, Supplemental Digital Content 3, http://links.lww.com/SCS/G245). Recurring opinions concerning care for FDS patients in the survey were formed into statements in discussion with the patient representatives from the expert panel statements were numbered throughout the voting rounds, and are referred to using these numbers between parentheses in the results section (Table 3, Supplemental Digital Content 3, http://links.lww.com/SCS/G245).

### Delphi Process

Health care providers with expertise in FDS from 13 centers affiliated with ERN CRANIO in 9 European countries were invited to participate in the Delphi voting process, along with 3 patient representatives. Experts were urged to only vote on statements related to their area of expertise. A 9-point Likert scale was used to measure agreement, from (1) “strongly disagree” to (9) “strongly agree.” Consensus on a statement was defined as a mean score of ≥7.00 and ≤1 outlier, where outliers were defined as any rating ≥2 Likert points away from the mean.^12^ To achieve consensus, a minimum of 5 votes per statement was required. A statement was considered “near consensus” with a mean score of ≥6.50 and/or ≤2 outliers.

In total, the Delphi process involved 3 voting rounds. Statements that were near consensus and remaining statements that were pointed out by members of the expert panel, were discussed after each round in plenary (online) sessions or in subgroups based on specialty. These statements were then refined or replaced with alternative ones by the expert panel for the subsequent voting round. After the final (third) voting round, no further adjustments or replacements were made.

## RESULTS

### Literature Search, Statement Formation, and Voting Process

**FIGURE 1:**
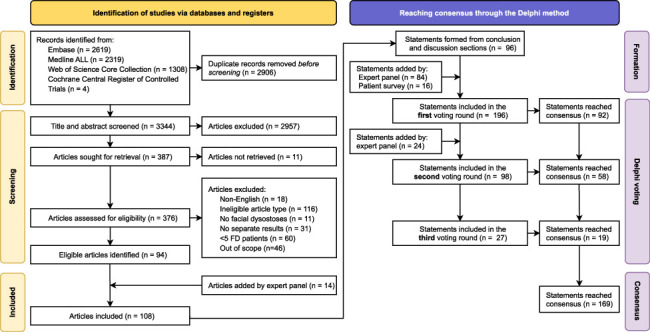
Flow chart of article selection and Delphi process.

Figure 1 shows the article selection and Delphi process. After deduplication and screening of the remaining 3344 articles, 94 met the eligibility criteria. Another fourteen articles were supplemented by the expert panel to fill evidence gaps. From these articles, 96 statements were derived, 84 were subsequently added by the panel based on their expert opinion, and 16 were added based on the patient survey (17 responses). For the second voting round, 24 additional statements were added by the expert panel to increase coverage, given the broad nature of FDS. In total, 196, 98, and 27 statements entered the first, second, and third voting rounds, of which 92 (46.9%), 58 (59.2%), and 19 (70.4%) reached consensus. Overall, 92 experts from various specialties and 3 patient representatives were involved in the voting process (Supplemental Digital Table 2, Supplemental Digital Content 1, http://links.lww.com/SCS/G243). In Table 3 (Supplemental Digital Table 3, Supplemental Digital Content 1, http://links.lww.com/SCS/G243),^19–36^ voting results and quality appraisal of source literature are presented for statements that reached a consensus. An overview of all statements, including those that did not reach a consensus, is provided in Supplemental Digital File 3, Supplemental Digital Content 4, http://links.lww.com/SCS/G246.

### General

Overall, 23 general statements about the care of FDS patients entered the Delphi process, of which 12 (52.2%) reached consensus (Supplemental Digital File 3, Supplemental Digital Content 4, http://links.lww.com/SCS/G246, nr. 1–22; 197). According to the experts, optimal care for FDS patients necessitates a multidisciplinary treatment team to ensure the best outcomes (2). This team ideally includes specialists who are knowledgeable about craniofacial reconstruction, upper airway management, the eyes and lacrimal system, speech, feeding, swallowing, dental treatment, orthodontics, hearing, extracranial anomalies, psychology, cognition, genetics, and coordination of care (3). When national expertise on these topics falls short, it can be helpful to contact experts within specialized networks, such as the ERN CRANIO, for adequate referral and treatment of the patient (18).

If FDS is detected prenatally, referral to a specialized obstetric unit is considered necessary to optimize the chances of survival during childbirth due to possible breathing problems (19). Besides, the referral of the parents to a multidisciplinary team is recommended by the experts for possible genetic consultation and to inform them about the condition and the patient’s journey (22b).

At the beginning of the care pathway, health care providers from the center of expertise should (again) provide parents with information on the anticipated consequences of the condition (7, 8). This also applies to FDS patients once they can understand. Simultaneously, health care providers should explain the organization of care for FDS patients (eg, multidisciplinary treatment team) or discuss alternatives (9). Moreover, both parents and patients should receive information on the tailored treatment plan and why it is needed throughout the treatment (5, 6). This includes providing information before each intervention, outlining its aim(s), postinterventional care, and possible complications, and allowing for questions (12). Finally, the experts agreed that contact among FDS patients may be helpful to them for social support and acceptance, for example, through patient organizations (16). Parents and patients should, therefore, ideally also be informed about these national and international organizations.

### Craniofacial Reconstruction

Regarding craniofacial reconstruction, a total of 62 statements entered the Delphi process, resulting in a consensus being achieved for 45 (72.6%) of them (Supplemental Digital File 3, Supplemental Digital Content 4, http://links.lww.com/SCS/G246, nr. 23–76, 198–205). Experts agreed that the treatment of craniofacial anomalies in FDS patients (ie, Treacher Collins, Nager, and Miller) may be approached in a similar manner (23). Nevertheless, they also agreed that functional problems and treatment outcomes in patients with Nager and Miller syndromes often require additional attention, given their frequently severe clinical presentations (24). Besides, the experts collectively expressed the necessity to discuss and plan craniofacial reconstruction for each FDS patient within a multidisciplinary treatment team setting, to ensure the best possible care (25). The optimal timing and sequence of craniofacial reconstruction procedures should be individualized based on the severity of the craniofacial anomalies and functional problems, including mouth opening, speech, feeding, (sleep related) breathing, closure of the eyelids, and hearing (26).^37^

Furthermore, to reliably evaluate craniofacial reconstruction procedures and their long-term effect, the experts agreed that 3D analysis techniques (ie, 3D photogrammetry, MRI, or CT) with combined soft tissue and skeletal modeling can be used (34). Future studies on craniofacial reconstruction in FDS should preferably incorporate these analysis techniques to accurately assess facial volume and shape changes (35). In addition, patient-reported outcome measures (PROMs) were considered appropriate for routine evaluations of satisfaction with appearance throughout treatment (38).

### Mandibular, Maxillary, and Malar Reconstruction

The experts also reached a consensus that FDS patients with maxillary and/or mandibular hypoplasia may benefit from counterclockwise correction (27). This approach enhances facial projection and appearance and may help in opening the posterior nasopharyngeal airway.^38,39^ Among the techniques to achieve this, the experts posed that counterclockwise distraction osteogenesis may provide more palatal rotation compared with other orthognathic surgeries (33).^40^ However, in cases where breathing issues is not of concern, it is preferred to carry out (final) orthognathic surgery once the patient reaches skeletal maturity to avoid unnecessary reoperation (28).^41^

It is also worth noting that surgical outcomes after mandibular distraction osteogenesis (MDO) should be assessed separately from other forms of micrognathia, such as Robin sequence (39). The success and complication rates with this procedure seem notably worse for FDS patients during growth.^42^ Therefore, the experts considered it important that the patient and their parent(s) are informed about all possible consequences of MDO (44).^43^

Furthermore, the experts concurred that combining orthognathic surgery with autologous fat transfer, either simultaneously or subsequently, could have a positive impact on the long-term facial contour and symmetry, besides function (ie, occlusion) (29).^44^ This could include lipofilling or dermal fat grafts, for instance of the suborbital region to reduce ectropion (30). Nonetheless, there is currently insufficient evidence on the relative effectiveness of different surgical reconstruction techniques of the orbitozygomatic and maxillary regions in FDS patients to allow for evidence-based recommendations (55).

In terms of the timing of orbitozygomatic reconstruction, the experts highlighted the need to consider the trade-off between short-term results on appearance and psychological well-being versus potentially restricted maxillary growth and the need for subsequent corrections, when contemplating this type of reconstruction before skeletal maturity (53). If opting for osseous orbitozygomatic reconstruction before skeletal maturity, delaying the procedure until the patient is 6 to 12 years old could reduce excessive bone resorption and the need for subsequent volumetric supplementation, while improving psychological well-being (54).^45^

### Temporomandibular Joint Reconstruction

Facial dysostosis syndrome patients with temporomandibular joint (TMJ) pathology (eg, ankylosis), in whom alternative treatments have proven ineffective or not feasible, may benefit from total prosthetic TMJ replacement to restore its function (47). If TMJ reconstruction is considered or when there are signs of abnormal TMJ function, such as restricted mouth opening, the use of 3D CT scanning of the zygomatic arch and TMJ region is helpful for informed decisions regarding the intervention (49).

### Eyelid Reconstruction

Regarding eyelid reconstruction, the experts agreed that, currently, there is insufficient evidence to determine the most effective surgical reconstruction techniques for eyelids (60). Notably, functional or esthetic abnormalities of the eyelids are often still present after eyelid surgery (61). However, the experts did agree that in the case of lower eyelid (pseudo)colobomas, an autologous (dermal) fat graft and subperiosteal malar lift with muscular or myocutaneous pedicled upper eyelid flaps may improve the configuration of the lower eyelids, considering position, tone, and volume (59). Besides, in patients with incomplete closure of the eyelids (lagophthalmos) and threatening exposure keratopathy, direct protective ointment with an eye bandage is required, possibly followed by eyelid surgery depending on the amount of corneal exposure (62). For patients with blepharoptosis or palpebral phimosis which could affect visual outcome, prompt corrective procedures may also be necessary according to the experts, followed by orthoptic follow-up to optimize visual outcomes (63, 64). Finally, the experts agreed that in FDS patients with eyelid abnormalities without functional consequences, there is no critical timing for reconstructive eyelid surgery (65), and the reconstruction is preferably done in a separate session after periorbital craniofacial surgery has been performed (66).

### External Ear Reconstruction

The experts concurred that external ear malformations in FDS patients can be treated according to the principles of the European guideline for craniofacial microsomia (58),^19^ as the ear malformations overlap. However, in FDS patients a possible lower hairline, temporal bone abnormalities, mandibular hypoplasia, hearing loss (treatment), and bilaterality of these problems should be considered in the planning. Therefore, functional ear surgery, including hearing implant placement, is preferably planned in consultation with the surgeon performing external ear reconstruction to ensure correct positioning (204).

Accordingly, FDS patients with external ear malformations may benefit from surgical reconstruction with synthetic implants or rib cartilage (198). In the case of previous unsuccessful reconstruction with rib cartilage or synthetic implants, osseointegrated implants are considered an appropriate alternative (203). Overall, the experts agreed that the choice between types of external ear reconstruction should be individualized for each patient, considering at least age, available tissue, local anatomy, presence of extracranial anomalies, and the preferences of parents and patients (199).

### Orofacial Cleft Repair

In the case of a cleft lip, the experts agreed that recommendations for the treatment of cleft lip from the guidelines on clefts of the lip and palate are appropriate for FDS patients (68).^46^ If a cleft palate is (also) present, its repair can be considered if the obstructive apnea-hypopnea index (AHI) is ≤5 and there is no significant carbon dioxide retention or polysomnography results with or without a custom-made palatal plate are normal (69). Once the patients undergo palate closure, a high-care spot should be readily available to monitor breathing (70) and parents should be informed that the closure may cause breathing problems requiring subsequent interventions (71). However, it should be noted that reliable evidence on the outcomes of palatal repair, including speech, feeding, and (nasal) breathing, in FDS patients with a cleft palate or palatal agenesis is currently lacking (72a). Similarly, evidence on the relative effectiveness of different surgical approaches for palate repair in FDS patients, including different timing, is also lacking (72b).

### Eyes and Lacrimal System

In total, 8 statements regarding the eyes and lacrimal system entered the Delphi process, and consensus was reached for 7 (87.5%) statements (Supplemental Digital File 3, Supplemental Digital Content 4, http://links.lww.com/SCS/G246, nr. 127–134). The experts came to an agreement regarding the importance of ophthalmologic screening in newly diagnosed FDS patients to prevent secondary anomalies and vision impairment. This initial screening ideally encompasses assessments for eyelid (pseudo)colobomas, lagophthalmos, and lacrimal drainage anomalies, as well as refractive errors, and strabismus, whenever possible, given their high prevalence (127).^2,47,48^ During follow-up examinations, the experts agreed that attention should be given to monitoring refractive errors and strabismus, and the possible presence of blepharoptosis, epiphora, exposure keratopathy, and amblyopia (128).^47,48^ Furthermore, at least one ophthalmologic evaluation after craniofacial surgery was considered beneficial for detecting and managing ocular anomalies that may arise due to surgical intervention (130).

Lastly, the experts agreed that management of lacrimal (drainage) anomalies should be tailored to the severity of epiphora, with conservative treatments for mild cases (132)^49^ and various interventional options for severe tearing depending on the cause, such as punctoplasty, balloon dacryoplasty, dacryocystorhinoplasty, or conjunctivodacryocystorhinostomy (133).

### Upper Airway

With respect to the upper airway, 39 statements entered the Delphi process, and consensus was reached for 33 (84.6%) statements (Supplemental Digital File 3, Supplemental Digital Content 4, http://links.lww.com/SCS/G246, nr. 77–108, 206–212). Given the frequent airway problems in FDS patients,^50–52^ the experts agreed that multidisciplinary evaluation is necessary before any airway intervention is undertaken to identify and address all levels of airway obstruction (78).^53,54^ In case of respiratory problems in the first year of life, a complete airway examination is necessary to rule out any associated abnormalities of the airway (79).

Furthermore, the experts collectively expressed that future studies on airway management in FDS patients should have a prospective design, standardized assessment and assessment ages, and comprehensive clinical outcomes (ie, polysomnography and upper airway endoscopy) to better understand the unique pathophysiology of airway problems in FDS patients (77).

### Obstructive Sleep Apnea (OSA)

The experts concurred that FDS patients may benefit from routine screening for OSA to enable timely diagnosis and treatment, given its high prevalence in both pediatric and adult patients (90).^50–52^ However, there is no consensus on the optimal frequency of OSA screenings in FDS patients (92). In case of OSA-related symptoms, such as breathing problems during sleep or abnormal growth curves, the experts did agree that a high frequency of screenings is necessary (91). Polysomnography is currently the gold standard to screen for OSA (83), while questionnaires such as the Epworth Sleepiness Scale and the Brouillette score were not considered appropriate (85).^55^

If left untreated, OSA may negatively affect the health-related quality of life due to frequent episodes of awakening during sleep and subsequent daytime sleepiness (96).^8^ Treatment for OSA is, therefore, preferable depending on the level(s) of obstruction. To determine the dynamics of the airway obstruction, airway endoscopy or Drug Induced Sleep Endoscopy (DISE) is helpful according to the experts (86, 88).^52,56^ In tracheostomized patients and patients with severe OSA, it is also considered appropriate to routinely perform these examinations (207). In these cases, the examinations are preferably performed simultaneously with other procedures, when possible, to minimize the need for multiple anesthesia exposures (208). If moderate or severe OSA is detected due to multilevel airway obstructions, the patient may benefit from noninvasive ventilation (eg, Continuous positive airway pressure or bilevel positive airway pressure) or a tracheostomy to relieve symptoms of obstructive breathing before interventions are undertaken to resolve OSA (94, 206).^52^

Ear, nose, and throat evaluation is preferable in case of (suspected) OSA to rule out adenoid and/or tonsillar hypertrophy (97), since FDS patients with enlarged tonsils or adenoids may benefit from (adeno)tonsillectomy (ATE) to reduce or resolve OSA (98). In efforts to resolve OSA, if ATE was not successful, mandibular distraction osteogenesis (MDO) may alleviate airway obstruction related to micrognathia (81).^54,57^ Airway endoscopy in combination with video-laryngoscopy and clinical examination is considered appropriate to determine MDO candidacy if severe OSA is present (104). Nevertheless, it is important to note that obstructions at all levels of the airway should be addressed in the total (surgical) treatment plan, to maximize the chances of resolving OSA (93). Moreover, recurrence of OSA after a successful MDO is still possible according to the experts and OSA screening remains necessary (95). To assess the airway outcomes after MDO in these patients, polysomnography at least 6 weeks after distraction is a reliable tool (89). When performing polysomnography in tracheostomized patients, it should be added that a closed cannula is necessary for reliable assessment of their airway patency (84).

### Intubation

With regard to anesthetic induction in FDS patients, the experts reached a consensus on the necessity of an airway management plan by the anesthesia team before every induction (99). In addition to this, adequately competent personnel and equipment are necessary to minimize the risk of airway complications. This is because FDS patients may require specialized intubation techniques, other than direct laryngoscopy, to secure the airway during surgery (101). However, if the intervention allows for it and endotracheal intubation is not necessary, a laryngeal mask may provide a good airway (102). When the patient is difficult to intubate, the experts also posed that a “difficult intubation ID card” can be helpful to inform health care providers in case of emergency (103).

### Tracheostomy and Decannulation

Some patients may require a tracheostomy to facilitate breathing. However, the average duration of tracheostomy requirement in tracheostomized patients remains undetermined at this point because of differences in underlying pathology (212). According to the experts, patients and/or parent(s) should, therefore, be informed that decannulation is not always possible (210). If decannulation is considered, patients and/or parent(s) should be informed that this is not always successful, and reinsertion may be necessary.

The experts concurred that decannulation of FDS patients can be considered if the cannula can be capped during the day and in case a sleep study in the hospital with a capped cannula and airway endoscopy do not reveal significant obstructions and only if the patient is discussed in a multidisciplinary team meeting (108a). During this meeting, it is necessary to at least discuss the safety of swallowing, outcomes of the sleep study and endoscopy (including the significance of remaining obstructions), ease of intubation, and the patient’s and parents’ preference concerning decannulation (108b).

### Genetics

Regarding genetics, 18 statements entered the Delphi process, and consensus was reached for 12 (66.7%) of them (Supplemental Digital File 3, Supplemental Digital Content 4, http://links.lww.com/SCS/G246, numbers 109–126). First, the experts reached a consensus on the need for referral of each patient with suspected FDS to a clinical geneticist for phenotypic evaluation and genetic counseling (109). Furthermore, genetic counseling should be offered to parents of FDS patients who are considering a new pregnancy (111). During this counseling, recurrence risk and options for prenatal testing and preimplantation genetic testing can be discussed (110). In the absence of a molecularly confirmed diagnosis of FDS, prenatal testing and preimplantation genetic testing are not possible (121), and in these cases, more extensive ultrasound(s) during pregnancy are preferably offered according to the experts (122). Moreover, when considering genetic counseling and reproductive options, possible somatic and germline mosaicism should be taken into account (124), since these mechanisms have been implied in the literature.^58^

Furthermore, the experts concurred that genetic diagnostics in patients with suspected FDS can be performed based on their phenotype and family history, after consent of the patient and/or parents (112). Also, targeted genetic testing is still indicated in family members of genetically confirmed patients, even in the absence of typical facial features of FD (125). This is due to the significant intrafamilial variability and reduced penetrance that have been reported in Treacher Collins Syndrome.^59^ However, it is important to note that the choice of genetic diagnostics largely depends on the lab facilities and the patient’s specific phenotypic presentation. Therefore, several methods can be used for genetic confirmation in FDS patients, for instance, a targeted gene panel approach (ie, TCOF1, POLR1B, POLR1C, POLR1D, SF3B4, DHODH)^4,60^ using Next Generation Sequencing including in silico gene panel analysis on a whole exome or genome sequencing platform, or in certain cases RNA-analysis (114, 115). Overall, targeted genetic analyses are preferred in patients with a phenotype strongly suggesting a specific FDS, and broader genetic analyses (eg, whole exome or genome sequencing) in those with atypical phenotypes (113) and in those left without genetic confirmation with older genetic analyses. Finally, in case of additional features suggesting other specific syndromes, a targeted gene approach or broader genetic testing (eg, multigene panel or whole exome or genome analysis) can also be indicated (116).

### Speech (Surgery)

Regarding speech and speech-enhancing surgery, 10 statements entered the Delphi process, and consensus was reached for all statements (Supplemental Digital File 3, Supplemental Digital Content 4, http://links.lww.com/SCS/G246, nr. 135–143, 213). The experts agreed that all newly diagnosed FDS patients may benefit from an evaluation of (pre-) speech and babbling skills (213), as well as a screening for speech problems at the beginning of speech (2 years) to allow for timely diagnosis and treatment (135). Subsequent standard evaluations of speech by a speech-language pathologist throughout treatment may be performed equivalently to patients with other craniofacial conditions, such as nonsyndromic cleft lip and palate (136). However, according to the experts, it should be pointed out that speech disorders in FDS patients may have multiple overlapping etiologies, such as a cleft palate, narrow upper airway, malocclusion, velopharyngeal insufficiency, a tracheostomy, and hearing loss, requiring careful differential diagnosis (137).^5^

The experts also concurred that, in the case of speech problems in FDS patients, speech therapy should preferably be started in consultation with the speech-language pathologist (ie, speech therapist) from the center of expertise (139). Under the supervision of the center of expertise, patients may receive speech therapy at their region of residence to reduce travel burden, besides their regular speech follow-up at the expert center (21a). Especially since FDS patients may require prolonged attention from speech-language pathologists into adulthood due to persistent speech problems (140).^61^

Facial dysostosis syndrome patients with speech problems due to velopharyngeal insufficiency may also benefit from double buccal myomucosal flaps as surgical treatment (143). Other surgical techniques can also be used; however, it is important that the risk of OSA is considered in the choice of surgery (142). To detect the problems with the mobility of the velopharyngeal sphincter, videofluoroscopy and nasoendoscopy are both considered helpful by the experts (138).

### Hearing

With respect to hearing, 27 statements entered the Delphi process, resulting in a consensus being achieved for 23 (85.2%) of them (Supplemental Digital File 3, Supplemental Digital Content 4, http://links.lww.com/SCS/G246, nr. 144–163, 214–220). The experts collectively expressed the necessity of audiological screening soon after birth to allow for timely treatment of hearing loss in FDS patients (146). They also agreed that otological and audiological screening at least every 3 years is beneficial for FDS patients to monitor hearing, irrespective of the severity of ear malformations (214).^62–64^ The reason for this is that FDS patients with minor ear malformations may still experience severe hearing loss and can benefit from hearing aids as early as possible (162).^63^

Moreover, the experts came to an agreement that air-conducting hearing devices are an appropriate first choice in FDS patients if acceptable hearing and speech intelligibility can be achieved, and their outer ear anatomy allows for wearing them (215). For FDS patients with malformations of the middle and/or outer ears resulting in conductive or mixed hearing loss, bone-anchored or bone-conducting hearing devices are appropriate alternatives (151).^20,63,65^ In these cases, implantable hearing devices are helpful if conventional hearing devices (eg, behind-the-ear devices, adhesive amplification, softband) are contra-indicated or are expected to give unsatisfactory results (152).^66,67^ Implantable devices are also considered helpful if the long-term outcomes of usual surgical intervention (ie, tympanoplasty, ear-canal meatoplasty, ossiculoplasty) are expected to be poor or reconstructive middle ear surgery is not feasible.^68^ Several implantable devices are appropriate in FDS patients according to the experts, including active middle ear implants (AMEI), percutaneous bone-conduction devices (BCD), transcutaneous passive BCDs, and transcutaneous active BCDs with an implanted actuator (144, 153).^20^ Supplemental Digital File 4, Supplemental Digital Content 5, http://links.lww.com/SCS/G247 provides an overview of the presently available hearing devices.

Overall, the choice of hearing device should be individualized for each patient, considering the type(s) of hearing loss, physical anatomy, and the preferences of the parent(s) and patient (216). Therefore, before surgery for hearing, health care providers should provide information on the different hearing devices, their principles of action, (dis)advantages, longevity, upgrade options, and MRI compatibility (145).^20^ Besides, the maximum power output (MPO) can be helpful for comparing and assessing hearing devices for the patient’s hearing function (149).^20^ To evaluate changes in hearing, audiological examinations before hearing intervention and 4 weeks after the acclimatization period are preferable (161, 220).

Lastly, the experts agreed that, currently, there is no conclusive evidence of the benefit of reconstructive middle ear or aural canal surgery to treat conductive hearing loss in FD patients (158).^62^ Therefore, bone-anchored or bone-conducting hearing aids are preferred over these types of reconstructive surgery for hearing rehabilitation (159).^63^ If reconstructive middle ear surgery is still considered, CT scanning of the temporal bones can be used to determine candidacy (157).

### Dental Treatment and Orthodontics

In total, 7 statements regarding dental treatment and orthodontics entered the Delphi process, and consensus was reached for 6 (85.7%) statements (Supplemental Digital File 3, Supplemental Digital Content 4, http://links.lww.com/SCS/G246, nr. 164–170). The experts came to an agreement about the benefit of biannual dental evaluations to prevent and treat oral diseases (164). The reasons for this is that FDS patients frequently show oral dryness caused by salivary gland pathology and mouth breathing, and often have a soft diet leading to a greater prevalence of dental tartar, cavities, and periodontal disease.^1,69,70^ Given the greater prevalence of dental anomalies,^71^ the experts also agreed on the benefit of at least one clinical and/or radiologic dental screening (168) and recommended that an orthodontist from the multidisciplinary team monitors the growth and development of the dentition and jaws (165). To reduce the travel burden, patients may receive treatment from an orthodontist in their own region of residence under the supervision of the center of expertise aside from orthodontic follow-up at the expert center (21b). Overall, FDS patients may require lifelong multidisciplinary dental care services to ensure optimal oral health and function, regardless of surgical interventions (167).

### Growth, Feeding, and Swallowing

Regarding growth, feeding, and swallowing, a total of 8 statements entered the Delphi process, resulting in a consensus being achieved for 6 (75.0%) of them (Supplemental Digital File 3, Supplemental Digital Content 4, http://links.lww.com/SCS/G246, nr. 171–178). The experts agreed that annual measurements of growth (ie, length and weight) are preferable in FDS patients between the ages of 1 to 6 years, to allow for timely diagnosis and treatment of growth disturbances (177). Obstructive sleep apnea and feeding problems should be considered as possible causes in case of insufficient growth in FDS patients (178). Besides growth disturbances, feeding problems also present a risk of aspiration and (near) fatal outcomes if left untreated. Therefore, the expert also reached consensus on the benefits of routine screening for feeding problems (174). In case of (suspected) feeding or swallowing problems, evaluation of feeding patterns by a speech therapist is preferable (175). When feeding problems are detected at an early age, feeding training may be beneficial and necessary, especially for FDS patients with a gastrostomy tube or tracheostomy (141).^72^ Nevertheless, multidisciplinary treatment strategies for feeding and swallowing problems in FDS patients should be further developed according to the experts (173).

### Extracranial Anomalies

With respect to extracranial anomalies, 8 statements entered the Delphi process, and consensus was reached for 5 (62.5%) statements (Supplemental Digital File 3, Supplemental Digital Content 4, http://links.lww.com/SCS/G246, nr. 179–186). In newly diagnosed FDS patients, the experts considered a comprehensive screening for extracranial anomalies preferable, with special attention to vertebral and cardiac anomalies, as well as limb anomalies in Nager and Miller syndrome, given their prevalence and clinical implications (182).^73–75^ Ideally, an examination for multiple vertebral anomalies covers spina bifida occulta, dysmorphic vertebrae and/or spinous process(es), reduced intervertebral space, scoliosis, and pectus excavatum or carinatum (183).^73,75^ Moreover, cardiac screening is preferably performed to detect anomalies including atrial and/or ventricular septal defect, and patent ductus arteriosus (184).^73,74^ According to the experts, it is paramount to inform parents and, when appropriate, FDS patients themselves about the potential existence of at least cardiac and vertebral anomalies, to allow for timely detection and management (186). Regarding patients with limb anomalies, the experts reached consensus on the importance of prompt referral to a specialized center to inform and guide parents (179).

### Psychology and Cognition

In total, 10 statements regarding psychology and cognition entered the Delphi process, and consensus was reached for 7 (70.0%) of them (Supplemental Digital File 3, Supplemental Digital Content 4, http://links.lww.com/SCS/G246, nr. 187–196). Starting with the parents and later including the patients, the experts considered psychological family consultations helpful in evaluating motivations, expectations, and the family’s planned involvement regarding reconstructive surgery, as well as coping style and resilience (187).^76^ Subsequently, parents of FDS patients should preferably have the opportunity to receive psychological and/or pedagogical support from a psychologist and/or social worker (193). This also includes offering prompt cognitive assessment by a qualified (neuro)psychologist if questions regarding the cognitive development of the child arise (190).

For FDS patients themselves, the combination of surgery and psychological support may enhance long-term psychological and social functioning according to the experts (188).^76–79^ To prepare young patients for (surgical) procedures, play therapy can be helpful (195). If the patient and/or their parent(s) suffer from anxiety at any point during treatment, assessment for psychological needs is preferable to allow for timely management (191). Similarly, if life-threatening situations occur at any point during treatment, psychological trauma in both the patient and parent(s) should specifically be screened for according to the experts (192).

## DISCUSSION

The goal of this clinical consensus statement was to provide statements reflecting expert consensus on the optimal diagnostic and treatment practices and research priorities in FDS care across Europe. While our efforts have yielded valuable insights and consensus on several key aspects, it is important to acknowledge the limitations and strengths of our approach.

One of the notable limitations of our study lies in the fact that respondents were urged to vote on statements within their perceived area(s) of expertise. Given the inherent differences in occupations of specialties across different countries and the complexity of the syndromes, this approach was essential to capture the entire range of perspectives. However, it introduced the possibility that some participants may have voted on statements beyond their primary area(s) of expertise. Despite this limitation, our study succeeded in collecting the wisdom of a diverse group of experts, including patient representatives. This contributes to a more comprehensive understanding of FDS management across various specialties and European countries from both patient and clinician perspectives.

A second limitation of our study was the small number of respondents for statements related to limb anomalies, with only 2 respondents providing input. This limitation underscores the difficulty in gathering comprehensive expert consensus for conditions involving this many specialties. Future research in this area may require targeted efforts to engage specialists with expertise in limb anomalies to provide more robust recommendations on this topic. Nevertheless, the substantial participation of health care providers from various other specialties remains a notable strength of this study. Despite the challenges of coordinating a large group of health care providers across Europe, considerable response rates were achieved in each of the 3 voting rounds (62.9%, 85.3%, 92.6%, Supplemental Digital Table 2, Supplemental Digital Content 1, http://links.lww.com/SCS/G243). This high level of engagement reflects the commitment of health care professionals and patient representatives across Europe to contribute to the development of comprehensive clinical recommendations for these syndromes.

Furthermore, it is crucial to note the specific challenge posed by Miller syndrome within our study. Unlike Treacher Collins and Nager syndromes, literature on Miller syndrome primarily comprises case reports and small case series, accounting for ∼40 cases in total. This scarcity of evidence not only highlights the need for a cautious interpretation of some of our recommendations for Miller syndrome, but also underscored a broader issue encountered in the field. Given that many advances in genetics are recent, such as the discovery of the causative DHODH gene for Miller syndrome around 2010^80,81^, misdiagnoses may be common in studies lacking genetic confirmations for FDS patients. The scarcity of evidence and likelihood of misdiagnoses both emphasize the necessity for further research on these syndromes with special attention to diagnostic accuracy. Nevertheless, given the overlap in phenotypical characteristics and treatments, our recommendations are still considered valuable for patients with Miller syndrome.

A final point of discussion included the strict consensus criteria that allowed for only one outlier response, making it challenging to reach a consensus on statements with numerous responses. However, the strict criteria enhance the credibility and reliability of the resulting statements, providing clinicians with trustworthy recommendations for the management of FDS. Hence, these strict criteria were rather considered a strength. Simultaneously, this resulted in a lack of consensus on some topics.

### Orbitozygomatic Reconstruction

One of the challenges encountered in our study was the lack of sufficient evidence to agree on the value of specific approaches for orbitozygomatic reconstruction. The field appears divided on this topic, with varying opinions and surgical approaches among experts. While some experts advocate for techniques employing (full thickness) bone grafts, others remain skeptical about their predictability and efficacy. Future research efforts should prioritize investigating these different approaches, seeking to provide clinicians with clearer recommendations on the most effective and stable ones.

### Tracheostomy Decannulation

The criteria for tracheostomy decannulation are another aspect that requires attention in future research. While limited agreement has been achieved on the overall criteria for decannulation in pediatric patients in previous studies,^82,83^ high levels of consensus were reached in this study (108a, 108b). Nevertheless, exact criteria still vary across centers. This variation in criteria can partly be attributed to differences in national organization of care. For instance, large traveling distances to the expert hospital may prevent decannulation in some countries, to ensure a safe airway in the region of residence in case of emergency. Hence, these criteria require targeted research to find the safest and most effective criteria in FDS, especially given their unique combination of airway obstructions at multiple levels.

### Feeding and Swallowing Problems

Currently, there is a notable scarcity of literature addressing feeding and swallowing problems in FDS, despite their substantial impact on patients and parent(s). Therefore, research on these topics is necessary, starting with the basis. This includes mapping the oral and pharyngeal phases of swallowing at various ages (eg, 0–3 months, 6 months, 15 months, 2 years) and assessing the risk of aspiration. In addition, there is a need for research on the effectiveness of MDO and prespeech feeding training in alleviating swallowing problems to allow for better decision-making and informing of parents. Lastly, there is a lack of objective examination regarding differences in the difficulty of consuming foods of different consistencies and possible underlying causes (eg, jaw anatomy, salivary secretion, breathing difficulties).

### Patient-Reported Outcome Measures

Although consensus was reached on the appropriateness of patient-reported outcome measures (PROMs) for routine evaluations of appearance by the patient throughout treatment (38a), there was no agreement on an appropriate frequency of evaluations. Besides, it is important to mention that some experts expressed the need to be careful with PROMs, especially novel ones, as their questions may be interpreted differently by patients depending on their condition or may be difficult to interpret at a younger age. Additional research on the use of PROMs and an appropriate frequency in patients with FDS is, therefore, also desirable.

### Developmental Risks

In patients with Treacher Collins, Nager, and Miller syndrome, there is no established direct causal association between the genetic background and the occurrence of cognitive and neurodevelopmental disabilities. However, these patients may face an increased risk of acquired neurodevelopmental disabilities. This elevated risk may largely be attributed to the prevalence of multiple concurrent challenges related to vision, hearing, speech, breathing, and digestion. While problems with vision, speech, and hearing pose a direct threat to neurodevelopment, problems with breathing (ie, OSA) and digestion (ie, gastroesophageal and laryngopharyngeal reflux) may indirectly affect development due to episodic hypoxia and sleep fragmentation.^84^ Subsequently, sleep fragmentation may lead to excessive daytime sleepiness resulting in a lack of concentration and a less efficient inquiry of knowledge.^8,84^ This complex interplay of factors highlights the importance of multidisciplinary management for FDS patients and the necessity for frequent screening to optimize their developmental progress and overall well-being. It also points out the need for well-designed (prospective) studies to better understand the magnitude of these risk factors and to optimize their management in the future.

In conclusion, our clinical consensus statement, developed through collaborative efforts of European experts within the ERN CRANIO network and patient representatives, provides valuable insights into the diagnostic and treatment practices for patients with FDS. Despite limitations, our study offers accurate insight into optimal practices for diagnosis and treatment, and research opportunities to improve care according to Europe’s experts in FDS. These research opportunities include orbitozygomatic reconstruction, tracheostomy decannulation criteria, feeding and swallowing problems, patient-reported outcome measures, and developmental risks. This consensus statement represents a significant step in advancing care for patients with FDS in Europe, emphasizing the ongoing commitment of health care professionals to improve understanding and management of these rare syndromes.

## Supplementary Material
