# Supplementary material for: Optimal Diagnostic and Treatment Practices for Facial Dysostosis Syndromes: A Clinical Consensus Statement Among European Experts
Source: J Craniofac Surg. 2024 May 27;35(5):1315–24. doi: 10.1097/SCS.0000000000010280 (PMC11198962; doi:10.1097/SCS.0000000000010280)
Supplement: Supplementary file 1 [file scs-35-1315-s001.docx]

| **Supplemental Digital Table 1 -** Expert panel members | | |
| --- | --- | --- |
| **Profession** | **Representative** | **Center** |
| Anesthesiologist | Anouar Bouzariouh | Erasmus Medical Center |
|  | Peter Frykholm | Uppsala University Hospital |
| Clinical geneticist | Marieke van Dooren | Erasmus Medical Center |
|  | Anne Goverde | Erasmus Medical Center |
|  | Alexandra Topa | Sahlgrenska University Hospital |
| ENT specialist / Otolaryngologist | Briac Thierry | Hôpital Universitaire Necker Enfants-Malades |
| Maxillofacial surgeon | Eppo Wolvius | Erasmus Medical Center |
|  | Víctor Zafra Vallejo | Hospital 12 de Octubre |
|  | Montserrat Munill | Vall d'Hebron |
| Nurse practitioner | Elin Weissbach | Erasmus Medical Center |
| Ophthalmologist | Sjoukje Loudon | Erasmus Medical Center |
|  | Eva Larsson | Uppsala University Hospital |
| Orthodontist | Stephen Tjoa | Erasmus Medical Center |
|  | Arja Heliovaara | Helsinki University Hospital |
| Pediatrician | Gwen van Heesch | Erasmus Medical Center |
|  | Koen Joosten | Erasmus Medical Center |
| Plastic surgeon | Christianne van Nieuwenhoven | Erasmus Medical Center |
|  | Sarah Versnel | Erasmus Medical Center |
|  | Irene Mathijssen | Erasmus Medical Center |
| Psychologist | Marizela Kljajic | Sahlgrenska University Hospital |
|  | Maria Valvassori | San Gerardo Hospital |
|  | Åsa Alberius Munkhammar | Uppsala University Hospital |
| Speech and Language Pathologist | Henriette Poldermans | Erasmus Medical Center |
|  | Pamela Marika Åsten | Oslo University Hospital |
| Patient representatives | Mariët Faasse | LAPOSA |
|  | Arnoud Heinen | LAPOSA |
|  | Ivana Marinac | Rare Diseases Croatia |

| **Supplemental Digital Table 2** – Characteristics of Delphi Respondents | | | | |
| --- | --- | --- | --- | --- |
|  | **Expert panel (n=28)*** | **First  round (n=95)** | **Second round (n=81)** | **Third round (n= 75)** |
| Number of invitations sent | - | 151 | 95 | 81 |
| Response rate | - | 62.9% | 85.3% | 92.6% |
|  |  |  |  |  |
| **Profession** |  |  |  |  |
| Anesthesiologist | 2 (7.1%) | 5 (5.3%) | 4 (4.9%) | 3 (4.0%) |
| Audiologist | 0 (0%) | 2 (2.1%) | 2 (2.5%) | 2 (2.7%) |
| Clinical geneticist | 4 (14.3%) | 7 (7.4%) | 6 (7.4%) | 6 (8.0%) |
| ENT specialist / Otolaryngologist | 1 (3.6%) | 9 (9.5%) | 7 (8.6%) | 7 (9.3%) |
| Maxillofacial surgeon | 3 (10.7%) | 12 (12.6%) | 11 (13.6%) | 10 (13.3%) |
| Nurse practitioner | 1 (3.6%) | 5 (5.3%) | 3 (3.7%) | 3 (4.0%) |
| Ophthalmologist | 2 (7.1%) | 5 (5.3%) | 4 (4.9%) | 4 (5.3%) |
| Orthodontist | 2 (7.1%) | 7 (7.4%) | 5 (6.2%) | 4 (5.3%) |
| Patient representative | 3 (10.7%) | 3 (3.2%) | 3 (3.7%) | 3 (4.0%) |
| Pediatrician | 2 (7.1%) | 6 (6.3%) | 6 (7.4%) | 5 (6.7%) |
| Plastic surgeon | 3 (10.7%) | 13 (13.7%) | 11 (13.6%) | 11 (14.7%) |
| Psychologist | 3 (10.7%) | 8 (8.4%) | 8 (9.9%) | 8 (10.7%) |
| Radiologist | 0 (0%) | 3 (3.2%) | 3 (3.7%) | 3 (4.0%) |
| Speech and Language Pathologist | 2 (7.1%) | 8 (8.4%) | 6 (7.4%) | 5 (6.7%) |
| Other | 0 (0%) | 2 (2.1%) | 2 (2.5%) | 1 (1.3%) |
|  |  |  |  |  |
| **Hospital (Country code)** |  |  |  |  |
| Erasmus Medical Centre (NL) | 13 (46.4%) | 24 (25.3%) | 21 (25.9%) | 19 (25.3%) |
| Great Ormond Street Hospital (UK) | 0 (0%) | 3 (3.2%) | 3 (3.7%) | 3 (4.0%) |
| Helsinki University Hospital (FIN) | 1 (3.6%) | 4 (4.2%) | 4 (4.9%) | 4 (5.3%) |
| Hôpital Universitaire Necker-Enfants Malades (F) | 1 (3.6%) | 8 (8.4%) | 6 (7.4%) | 5 (6.7%) |
| Hospital 12 de Octubre (E) | 1 (3.6%) | 7 (7.4%) | 7 (8.6%) | 6 (8.0%) |
| Hospital de Santa Maria (P) | 0 (0%) | 3 (3.2%) | 1 (1.2%) | 0 (0%) |
| Oslo University Hospital (N) | 1 (3.6%) | 5 (5.3%) | 4 (4.9%) | 4 (5.3%) |
| Sahlgrenska University Hospital (S) | 2 (7.1%) | 9 (9.5%) | 5 (6.2%) | 5 (6.7%) |
| San Gerardo Hospital (I) | 1 (3.6%) | 3 (3.2%) | 2 (2.5%) | 2 (2.7%) |
| Smile House - San Paolo Hospital (I) | 0 (0%) | 3 (3.2%) | 4 (4.9%) | 4 (5.3%) |
| University Medical Centre Utrecht (NL) | 0 (0%) | 8 (8.4%) | 8 (9.9%) | 7 (9.3%) |
| Uppsala University Hospital (S) | 4 (14.3%) | 10 (10.5%) | 8 (9.9%) | 8 (10.7%) |
| Vall d'Hebron Hospital (E) | 1 (3.6%) | 5 (5.3%) | 5 (6.2%) | 5 (6.7%) |
|  |  |  |  |  |
| Patient organizations  (LAPOSA, Rare Diseases Croatia) | 3 (10.7%) | 3 (3.2%) | 3 (3.7%) | 3 (4.0%) |
|  |  |  |  |  |

*All members of the expert panel completed a European Committee declaration of interest form for taskforce members of European Reference Networks guidelines. No conflicting interests were noted. All completed forms are available at the corresponding author upon request.

| **Supplemental Digital Table 3** - Overview of statements that reached consensus | | | | | | |
| --- | --- | --- | --- | --- | --- | --- |
| **Statement** | **Mean (outliers)** | **Number of votes** | **Level of evidence*** | **Quality level*** | **First author (year)** | **Ref.** |
| ***General*** |  |  |  |  |  |  |
| 2. A multidisciplinary treatment team is necessary to achieve optimal care in FDS patients. | 8.7 (1) | 92 | V | - | - | - |
| 3. A treatment team for FDS patients ideally includes specialists who are knowledgeable about craniofacial reconstruction, upper airway management, the eyes and lacrimal system, speech, feeding, swallowing, dental treatment, orthodontics, hearing, extracranial anomalies, psychology and cognition, genetics, and coordination of care. | 8.3 (1) | 72 | V | - | - | - |
| 5&6. Throughout treatment, health care providers from the center of expertise should provide parents and patients with information on the tailored treatment plan and why it is needed (e.g. face-to-face, digitally, brochures) | 8.7 (0) | 74 | V | - | - | - |
| 7&8. Health care providers from the center of expertise should provide parents with information on the foreseen consequences of the condition and prognosis, at the beginning of the treatment journey, and the FDS patient as soon as they are able to understand. (e.g. digitally or brochures) | 8.3 (1) | 76 | V | - | - | - |
| 9. At the beginning of the treatment journey, health care providers should provide information on the organisation of care regarding the FDS patient (e.g. multidisciplinary treatment team), or discuss alternatives (e.g. referral to expert center) | 8.6 (0) | 74 | V | - | - | - |
| 12. Before each intervention, health care providers should provide information on the intervention, its aim(s), post-interventional care, and possible complications, as well as the opportunity to ask questions. | 8.8 (0) | 92 | V | - | - | - |
| 16. Contact amongst FDS patients, for example through patient organizations, may be helpful for social support and acceptance. | 8.6 (1) | 79 | V | - | - | - |
| 18. If there is insufficient expertise on (aspects of) the treatment of FDS patients nationally, contacting experts within specialized healthcare networks as a healthcare provider, such as the ERN CRANIO, can be helpful for adequate referral and/or treatment of the FDS patient. | 8.6 (1) | 77 | V | - | - | - |
| 19. In case of prenatal detection of FDS, referral to the designated obstetric unit with standby pediatric anesthesiologists and surgeons specialized in difficult airway management is necessary to optimize chances of survival during childbirth. | 8.6 (1) | 68 | V | - | - | - |
| 21a. Under supervision of the center of expertise FDS patients may, besides their regular speech follow-up at the expert center, receive speech therapy in their own region of residence to reduce travel burden for parents. | 8.6 (1) | 68 | V | - | - | - |
| 21b. Under the supervision of the center of expertise FDS patients may receive treatment by an orthodontist in their own region of residence, besides orthodontic follow-up at the expert center | 8.6 (1) | 53 | V | - | - | - |
| 22b. In case of prenatal suspicion of FDS, referral to a multidisciplinary expert team is recommended for possible genetic confirmation and to inform parent(s) about the condition and patient's journey. | 8.5 (1) | 65 | V | - | - | - |
|  |  |  |  |  |  |  |
| ***Craniofacial Reconstruction*** |  |  |  |  |  |  |
| 23. The treatment of craniofacial anomalies in Treacher Collins, Nager, and Miller syndrome may be approached in a similar manner. | 7.9 (1) | 21 | V | - | - | - |
| 24. Functional problems and treatment outcomes related to craniofacial anomalies in Nager and Miller syndromes often require additional attention. | 7.8 (1) | 24 | V | - | - | - |
| 25. Discussions and planning of craniofacial reconstruction within a multidisciplinary team is necessary to achieve optimal care in FDS patients. | 8.8 (1) | 33 | V | - | - | - |
| 26. The optimal timing and sequence of craniofacial reconstruction procedures in FDS patients should be individualized based on the severity of the craniofacial anomalies and functional problems, including problems with speech, feeding, (sleep-related) breathing, closure of the eyelids, and hearing. | 8.8 (1) | 33 | III | Low | Plomp (2016) | ^19^ |
| 27. FDS patients with maxillary and/or mandibular hypoplasia may benefit from counterclockwise surgical correction of the maxilla and/or mandible in opening the posterior nasopharyngeal airway and improve facial projection and appearance. | 8.1 (0) | 22 | III | High | Freihofer (1997); Nguyen (2016) | ^20,21^ |
| 28. In the absence of breathing difficulties, it is preferred to perform final orthognathic surgery from skeletal maturity onwards. | 8.5 (0) | 25 | IV | Low | Roncevic (1996) | ^23^ |
| 29. Orthognathic surgery with simultaneous or subsequent autologous fat transfer may improve long-term facial contour, symmetry, and function (e.g. occlusion) in FDS patients. | 8.0 (1) | 22 | III | High | Lim (2012) | ^26^ |
| 30a. FDS patients may benefit from lipofilling of the lower eyelid and zygomatic area at a young age to reduce ectropion and improve symmetry and contour. | 8.3 (0) | 19 | V | - | - | - |
| 30b. Lipofilling or dermis fat grafts are helpful to correct the facial contour and symmetry for FDS patients | 8.3 (1) | 21 | V | - | - | - |
| 33. Counterclockwise craniofacial distraction osteogenesis may provide greater palatal rotation compared to other orthognathic operations in FDS patients. | 7.4 (1) | 19 | IV | High | Hopper (2018) | ^22^ |
| 34. 3D analysis techniques, such as 3D photogrammetry, CT or MRI, with combined soft and skeletal tissue modelling can be utilized for reliable evaluation of craniofacial reconstruction procedures and their long-term effects in FDS patients. | 8.3 (1) | 24 | V | Average | Ibrahim (2016) | ^69^ |
| 35. Future studies on craniofacial reconstruction of FDS patients should incorporate 3D analysis techniques, such as 3D photogrammetry, CT or MRI, to accurately assess changes in facial volume and shape. | 8.5 (1) | 26 | III | High | Apolloni (2020);  Lim (2012) | ^26,70^ |
| 38. Patient Reported Outcome Measures (PROMs) are appropriate for routine evaluations of appearance throughout treatment in FDS patients. | 8.2 (1) | 25 | V | - | - | - |
| 39. Surgical outcomes, including success rate and complications, after mandibular distraction osteogenesis (MDO) for FDS patients should be approached separately from other forms of micrognathia, such as Pierre Robin Sequence. | 8.0 (1) | 23 | IV | Average | Ali-Khan (2018) | ^24^ |
| 42. In FDS patients, mandibular distraction osteogenesis may compromise the development of the primary and/or secondary dentition, depending on the dental stage. | 7.7 (0) | 22 | IV | Average | Kleine-Hakala (2007) | ^71^ |
| 44. FDS patients (if possible) and their parents should be informed of all possible consequences of mandibular distraction, including pain, functional disturbances in jaw movement and dietary problems, possibility for decannulation, and nerve hypoaesthesia and paresis. | 8.8 (0) | 25 | IV | Average | Hurmerinta (2004) | ^25^ |
| 47. FDS patients with temporomandibular joint (TMJ) pathology, in whom other treatments have not been effective or are not deemed possible, may benefit from total prosthetic TMJ replacement to restore its function. | 7.7 (1) | 21 | V | Low | Schlieve (2012) | ^72^ |
| 48. 3D CT scanning is a reliable modality for the visualization of abnormalities in the zygomatic arch in FDS patients. | 8.6 (1) | 26 | IV | Average | Kaga (2003) | ^73^ |
| 49. 3D CT scanning of the zygomatic arch and temporomandibular joint (TMJ) is helpful in FDS patients before TMJ reconstruction or in case of abnormal TMJ function (e.g. restricted mouth opening). | 8.3 (0) | 24 | V | - | - | - |
| 53. The trade-off between short-term results on appearance and psychologic well-being versus stunted maxillofacial growth and need for subsequent corrections should be taken into account when considering orbitozygomatic reconstruction before skeletal maturity in FDS patients. | 7.6 (1) | 25 | V | - | - | - |
| 54. When opting for osseous orbitozygomatic reconstruction before skeletal maturity in FDS patients, postponement until 6-12 year may reduce excessive bone resorption and the need for subsequent volumetric supplementation, while improving psychologic well-being. | 7.5 (0) | 20 | IV | High | Fan (2012) | ^27^ |
| 55. Currently there is insufficient evidence on the relative effectiveness of different surgical reconstruction techniques (e.g. synthetic vs autologous) of the orbitozygomatic and maxillary regions in FDS patients. | 7.5 (0) | 22 | V | - | - | - |
| 58. External ear malformations in FDS patients, similar to craniofacial microsomia, can be treated according to the principles of the European Guideline Craniofacial Microsomia, although possible lower hairline, temporal bone abnormalities, mandibular hypoplasia, hearing loss (treatment), and bilaterality of these problems should be considered in the planning. | 8.3 (0) | 26 | V | N/A | Renkema (2020) | ^74^ |
| 198. FDS patients with external ear malformations may benefit from surgical reconstruction with synthetic implants (e.g. Medpore) or rib cartilage as surgical treatment. | 7.8 (1) | 14 | V | - | - | - |
| 199. The choice between types of external ear reconstruction should be individualized for each patient, considering (at least) age, available tissue, local anatomy, presence of extracranial anomalies, and the preferences of parents and patients | 8.4 (1) | 11 | V | - | - | - |
| 200. If rib cartilage is chosen for external ear reconstruction in FDS patients, reconstruction is preferably performed at eight years or older. | 8.1 (1) | 14 | V | N/A | Renkema (2020) | ^74^ |
| 203. In FDS patients with previous unsuccessful external ear reconstruction with rib cartilage or synthetic implants, auricular osseo-integrated implants are an appropriate alternative. | 7.9 (1) | 14 | V | N/A | Renkema (2020) | ^74^ |
| 204. Functional ear surgery, including hearing implant placement, is preferably planned in consultation with the surgeon performing external ear reconstruction to ensure correct positioning. | 8.9 (0) | 10 | V | - | - | - |
| 59. Autologous (dermal) fat graft and subperiosteal malar lift with (muscular or myocutaneous) pedicled upper eyelid flaps may improve the configuration of the lower eyelids in FDS patients, considering the position, tone, and volume of the lower eyelids. | 7.2 (1) | 17 | V | Average | Franchi (2016) | ^75^ |
| 60. Currently, there is insufficient evidence on the relative effectiveness of different surgical reconstruction techniques of the eyelids in FDS patients. | 7.7 (0) | 21 | V | - | - | - |
| 61. Reconstructive surgery can improve eyelid anomalies in FDS patients, but functional and/or aesthetic abnormalities of the eyelids are often still present after eyelid corrections. | 8.2 (0) | 14 | V | - | - | - |
| 62a. In FDS patients with incomplete closure of the eyelids (lagophthalmos) and threatening exposure keratopathy, direct protective eye ointment combined with an eye pad/shield/bandage is required. | 8.7 (0) | 14 | V | - | - | - |
| 62b. FDS patients with incomplete closure of the eyelids (lagophthalmos) may benefit from reconstructive eyelid surgery depending on the amount of corneal exposure | 8.5 (0) | 11 | V | - | - | - |
| 63. FDS patients with ptosis and threatening amblyopia, may benefit from ptosis correction at short notice and require orthoptic follow-up. | 8.0 (1) | 20 | V | - | - | - |
| 64. FDS patients with palpebral phimosis and a visual impairment, may benefit from reconstructive eyelid surgery at short notice | 7.7 (1) | 15 | V | - | - | - |
| 65. For FDS patients with eyelid abnormalities without functional consequences, there is no critical timing for reconstructive eyelid surgery. | 7.9 (1) | 13 | V | - | - | - |
| 66. For FDS patients with eyelid abnormalities without functional consequences, eyelid reconstruction is preferably performed in a separate session after peri-orbital craniofacial surgery has been performed. | 8.2 (1) | 11 | V | - | - | - |
| 68. Recommendations for the treatment of cleft lip from the guidelines on clefts of the lip and palate are appropriate for FDS patients. | 7.7 (1) | 18 | V | N/A | Mink van der Molen (2021) | ^29^ |
| 69. Cleft palate repair in FDS patients can be considered if the obstructive apnea-hypopnea index (AHI) is ≤ 5 and there is no significant carbon dioxide retention and/or polysomnography are normal with or without a custom-made palatal plate (if the hard palate is open). | 8.1 (1) | 16 | V | N/A | PRS Guideline |  |
| 70. A high care spot to monitor breathing should be readily available for FDS patients undergoing cleft palate repair, especially in case of previous abnormal polysomnography results. | 8.7 (1) | 21 | V | - | - | - |
| 71. Patients and/or their parents should be informed that closure of the cleft palate may cause breathing problems requiring subsequent interventions, such as re-opening the palate, if these breathing problems persist. | 7.8 (1) | 20 | V | - | - | - |
| 72a. Reliable evidence on the outcomes of palatal cleft closure in FDS patients, including speech, feeding, and (nasal) breathing, is currently lacking. | 7.7 (0) | 16 | V | - | - | - |
| 72b. Currently, evidence on the relative effectiveness of different surgical approaches/techniques for cleft palate repair in FDS patients is lacking | 8.1 (1) | 14 | V | - | - | - |
| 73. Structured physical nasal examination with nasal endoscopy is helpful to determine aspects that require more attention during nasal reconstructive surgery in FDS patients to optimize functional outcomes. | 7.9 (0) | 25 | III | Average | Plomp (2015) | ^76^ |
| 74. (Septo)rhinoplasty at adult age can lead to satisfactory results in FDS patients with considerable esthetic wish and/or nasal obstruction combined with septal deviation and/or nasal valve insufficiency. | 7.9 (0) | 23 | III | Average | Plomp (2015) | ^76^ |
|  |  |  |  |  |  |  |
| **Upper Airway** |  |  |  |  |  |  |
| 77. Future studies on airway management in FDS patients should have a prospective design, standardized assessment and assessment ages, and comprehensive clinical outcomes (i.e., polysomnography and upper airway endoscopy) to better understand the unique pathophysiology of airway problems in FDS patients. | 8.5 (1) | 24 | IV | Average | Ali-Khan (2018); Hosking (2012) | ^24,77^ |
| 78. Multidisciplinary evaluation is necessary to identify and address all levels of airway obstruction (choanae, pharynx, larynx) in FDS patients before any airway intervention is undertaken. | 8.6 (0) | 27 | IV | Average | Biskup (2018);  Tahiri (2014) | ^36,37^ |
| 79. In case of respiratory problems in the first year of life, a complete examination of the airway is necessary to rule out associated malformations | 8.7 (1) | 26 | V | - | - | - |
| 81. Mandibular lengthening distraction osteogenesis (MDO) may alleviate upper airway obstruction related to micrognathia in FDS patients. | 7.7 (0) | 18 | IV | Average | Tahiri (2014);  Van der Plas (2021) | ^37,40^ |
| 83. Polysomnography is currently the golden standard for screening OSA in FDS patients. | 8.4 (1) | 25 | V | - | - | - |
| 84. For reliable assessment of airway patency in tracheostomized FDS patients, a closed cannula is necessary during polysomnography. | 8.3 (1) | 22 | V | - | - | - |
| 85. The Epworth Sleepiness Scale and the Brouillette score do not accurately screen for OSA in FDS patients | 7.6 (0) | 14 | II | Average | Plomp (2012) | ^38^ |
| 86. Endoscopy of the upper airway may help determine the level and mechanism of airway obstruction in FDS patients with OSA. | 8.0 (0) | 26 | III | Average | Plomp (2012);  Sher (1986) | ^35,39^ |
| 88. DISE (Drug Induced Sleep Endoscopy) is helpful to determine the level and mechanism of airway obstruction in FDS patients with OSA. | 8.4 (1) | 22 | V | - | - | - |
| 89. Polysomnography at least six weeks after distraction, is a reliable tool to measure airway outcomes after mandibular and/or maxillary lengthening distraction in FDS patients. | 7.9 (1) | 19 | V | - | - | - |
| 90. FDS patients may benefit from routine screening for OSA to enable timely diagnosis and treatment. | 8.5 (0) | 25 | III | High | Moraleda-Cibrian (2014); Plomp (2012) | ^34,38^ |
| 91. A higher frequency of examinations of OSA are necessary in case of OSA-related symptoms, such as breathing problems during sleep or abnormal growth curves. | 8.4 (1) | 22 | V | - | - | - |
| 92. Currently, there is no evidence available on the optimal frequency for OSA screenings in FDS patients. | 8.2 (1) | 18 | V | - | - | - |
| 93. To maximize chances of resolving OSA, obstructions at all levels of the airway should be addressed in the (surgical) treatment of the FDS patient. | 8.4 (0) | 25 | V | - | - | - |
| 206. In FDS patients with severe OSA, a tracheostomy may be indicated when non-invasive measures to facilitate breathing give insufficient improvement of breathing. | 8.6 (1) | 23 | V | - | - | - |
| 94. FDS patients with moderate or severe OSA due to multi-level airway obstructions may benefit from non-invasive ventilation (e.g. CPAP or bilevel positive airway pressure), or a tracheostomy to facilitate breathing. | 8.3 (0) | 23 | III | Average | Plomp (2012) | ^35^ |
| 95. Recurrence of OSA after successful mandibular lengthening distraction osteogenesis (MDO) is still possible and, therefore, routine screening remains necessary. | 8.5 (1) | 22 | V | - | - | - |
| 207. Routine examinations of the entire airway using endoscopy is appropriate in tracheostomized FDS patients and FDS patients with severe OSA to keep assessing all levels of airway obstruction. | 8.2 (1) | 21 | V | - | - | - |
| 208. If tracheostomized FDS patients or FDS patients with severe OSA undergo surgery, it is preferable to perform endoscopic (routine) examinations of the airway simultaneously to minimize the need for multiple anesthesia exposures. | 8.5 (1) | 27 | V | - | - | - |
| 96. If left untreated, OSA may negatively affect health related QoL in FDS patients, related to episodes of awakening, subjective snoring, and daytime sleepiness. | 8.6 (0) | 23 | IV | High | Geirdal (2013) | ^8^ |
| 97. In case of (suspected) OSA, ENT evaluation is preferable to rule out adenoid and tonsillar hypertrophy in FDS patients. | 8.8 (0) | 24 | V | - | - | - |
| 98. FDS patients with enlarged tonsils and/or adenoids may benefit from (adeno)tonsillectomy to reduce or resolve upper airway obstruction (OSA). | 8.6 (1) | 24 | V | - | - | - |
| 99. Before every induction in a FDS patient, an airway management plan made by the anesthesia team is necessary, as well as adequately competent personnel and equipment, to minimize risk of airway complications. | 8.5 (1) | 25 | V | - | - | - |
| 101. To secure their airway during surgery, FDS patients may require specialized intubation techniques, other than direct laryngoscopy, by a (pediatric) otolaryngologist or anesthesiologist experienced with difficult pediatric airways. | 8.8 (1) | 23 | IV | Average | Hosking (2012); Sinkueakunkit (2013) | ^77,78^ |
| 102. If the intervention allows for it and endotracheal intubation is not necessary, a laryngeal mask may provide a good airway in FDS patients undergoing an intervention. | 8.4 (1) | 21 | V | Average | Hosking (2012) | ^77^ |
| 103. In case of difficult intubation in FDS patients, a "difficult intubation ID card" can be helpful to inform healthcare providers in case of emergency. | 8.5 (0) | 22 | V | - | - | - |
| 104. Preoperative airway endoscopy in combination with video-laryngoscopy and clinical examination are helpful to determine distraction osteogenesis candidacy in FDS patients. | 7.8 (0) | 17 | V | - | - | - |
| 105. Preoperative airway endoscopy alone is not an appropriate predictor for successful decannulation after distraction osteogenesis (DO) and should therefore not be the sole determinant for DO candidacy in FDS patients. | 7.9 (1) | 21 | IV | Average | Sorin (2004) | ^79^ |
| 106. Mandibular distraction osteogenesis does not guarantee the possibility of decannulation in FDS patients. | 8.3 (0) | 23 | IV | Average | Sorin (2004) | ^79^ |
| 209. Patients and/or their parents should be informed that decannulation is not always successful and tracheostomy tube reinsertion may be necessary. | 8.8 (0) | 25 | V | - | - | - |
| 210. Patients and/or their parents should be informed that decannulation is not always possible in tracheostomized FDS patients. | 8.5 (1) | 26 | V | - | - | - |
| 212. The average duration of tracheostomy requirement in tracheostomized FDS patients remains undetermined | 8.5 (1) | 27 | V | - | - | - |
| 108a. Decannulation of FDS patients can be considered if the cannula can be capped during the day, a sleep study in the hospital with capped cannula and airway endoscopy do not reveal significant obstructions and additional factors and concerns that influence decannulation are discussed in the multidisciplinary team. | 8.6 (1) | 24 | V | - | - | - |
| 108b. In a multidisciplinary team meeting to evaluate possibility to decannulate, it is necessary to at least discuss: safety of swallowing, outcomes of the sleep study and endoscopy including significance of remaining obstructions, ease of intubation, and the patient's and parents' preference | 8.5 (0) | 26 | V | - | - | - |
|  |  |  |  |  |  |  |
| **Genetics** |  |  |  |  |  |  |
| 109. Referral of each patient with suspected FDS to a clinical geneticist is necessary for dysmorphic evaluation and genetic counseling. | 8.4 (1) | 14 | V | - | - | - |
| 110. Genetic counseling for the parents of a patient with genetically confirmed FDS is preferably offered, in which recurrence risk and options for prenatal testing and pre-implantation genetic testing are discussed. | 8.7 (0) | 14 | V | - | - | - |
| 111. Offering genetic counseling is appropriate in parents of an FDS patients with a wish for another child. | 8.8 (0) | 15 | V | - | - | - |
| 112. Genetic diagnostics in a patient with suspected FDS can be performed based on phenotype and family history, after consent of the FDS patient and parents | 8.8 (0) | 11 | V | - | - | - |
| 113a. In patients with a phenotype strongly suggesting a specific FDS syndrome, a targeted genetic analysis is preferred over a broader analysis (the choice for analytical method may depend on local policies or laboratory facilities) | 7.4 (1) | 8 | V | - | - | - |
| 113b. In patients with a phenotype atypical for FDS syndromes, broader genetic analyses (e.g. whole exome or genome sequencing) are preferred over targeted analyses (the choice for analytical method may depend on local policies or laboratory facilities) | 8.0 (1) | 8 | V | - | - | - |
| 114. Next Generation Sequencing targeted gene panel approach (TCOF1, POLR1B, POL1C, and POL1D) can be used for genetic confirmation in FDS patients. | 8.8 (0) | 8 | V | Average | Bukowska-Olech (2020); Pan (2021) | ^4,43^ |
| 115. Whole genome sequencing and/or RNA analyses after negative routine DNA testing, can be used for genetic confirmation in FDS patients | 8.0 (1) | 7 | V | - | - | - |
| 116. In case of additional features in FDS patients, suggesting specific other syndromes, a targeted gene approach or broader genetic testing, such as multigene panel, can be performed. | 9.0 (0) | 9 | V | - | - | - |
| 121. In case of unconfirmed genetic diagnosis of FDS, prenatal testing and pre-implantation genetic testing are not possible | 8.2 (1) | 10 | V | - | - | - |
| 122. In case of unconfirmed genetic diagnosis of FDS, additional ultrasound(s) during pregnancy are preferably offered | 7.9 (1) | 10 | V | - | - | - |
| 124. Somatic and germline mosaicism should be taken into account when considering genetic counseling and reproductive options in FDS patients. | 8.6 (1) | 7 | V | Average | Chen (2018) | ^41^ |
| 125. Targeted genetic testing is still indicated in family members of genetically confirmed FDS patients, even if they have no facial features of FDS. | 8.2 (1) | 9 | IV | Average | Teber (2004) | ^42^ |
|  |  |  |  |  |  |  |
| **Eyes and Lacrimal System** |  |  |  |  |  |  |
| 127. Ophthalmologic screening in newly diagnosed FDS patients should ideally cover at least eyelid (pseudo)colobomas, lagophthalmos, inferior punctal agenesis and other lacrimal drainage anomalies, as well as refractive errors and strabismus if possible. | 8.6 (1) | 7 | IV | High | Ali (2017);  Hertle (1993);  Rooijers (2022) | ^2,30,31^ |
| 128. Ophthalmologic follow-up examinations in FDS patients should ideally cover at least blepharoptosis, epiphora, exposure keratopathy, and amblyopia, as well as refractive errors and strabismus. | 8.4 (1) | 9 | IV | High | Hertle (1993);  Rooijers (2022) | ^30,31^ |
| 130. FDS patients may benefit from at least one ophthalmologic examination after craniofacial surgery. | 8.6 (0) | 7 | V | - | - | - |
| 131. Ophthalmologic follow-up is necessary in FDS patients with any kind of ocular anomaly with a frequency depending on the type(s) and most vision-threatening anomaly | 8.6 (0) | 9 | V | - | - | - |
| 132. Lacrimal anomalies in young FDS patients with only mild intermittent tearing (epiphora) may be treated conservatively. | 7.9 (1) | 7 | III | Average | Bartley (1990) | ^32^ |
| 133. In case of severe tearing (epiphora) in FDS patients, several treatments can be considered depending on the cause, such as punctoplasty, balloon dacryoplasty, dacryocystorhinoplasty, and conjunctivodacryocystorhinostomy. | 7.6 (0) | 8 | V | - | - | - |
| 134. Amblyopia in FDS patients can be treated in the same manner as for other (non-syndromic) patients. | 8.2 (1) | 5 | V | - | - | - |
|  |  |  |  |  |  |  |
| **Speech (Surgery)** |  |  |  |  |  |  |
| 135. FDS patients may benefit from screening for speech problems at the beginning of speech (~2 year) to allow for timely diagnosis and treatment | 8.4 (1) | 17 | V | - | - | - |
| 136. Standard evaluation of speech by a speech language pathologist throughout treatment, may be performed in a manner equivalent to that used for patients with other craniofacial anomalies, such as a cleft lip and palate. | 8.0 (0) | 16 | V | - | - | - |
| 137. Speech disorders in FDS patients may have multiple overlapping etiologies, such as palatal cleft, malocclusion, velopharyngeal insufficiency, and a tracheostomy requiring careful differential diagnosis. | 8.5 (1) | 19 | III | Average | Vallino-Napoli (2002) | ^5^ |
| 138. Video-fluoroscopy and nasoendoscopy are helpful to detect problems with the mobility of the velopharyngeal sphincter in FDS patients with speech problems. | 8.3 (1) | 18 | V | - | - | - |
| 139. In case of speech problems in FDS patients, speech therapy should preferably be started in consultation with the speech-language pathologist part of the multidisciplinary team from the center of expertise. | 8.4 (1) | 19 | V | - | - | - |
| 140. FDS patients may require prolonged attention of speech-language pathologists into adulthood due to persistent speech problems. | 8.3 (0) | 19 | III | High | Åsten (2014) | ^44^ |
| 141. Pre-speech feeding training may be beneficial for FDS patients, especially in patients with gastrostomy tubes or tracheostomy. | 8.3 (0) | 14 | IV | Low | Meyerson (1987) | ^56^ |
| 142. The risk of OSA should be considered in the choice of surgery for the treatment of velopharyngeal insufficiency in FDS patients. | 8.6 (1) | 19 | V | - | - | - |
| 143. FDS patients with velopharyngeal insufficiency may benefit from double buccal myomucosal flaps as surgical treatment | 7.5 (0) | 11 | V | - | - | - |
| 213. Evaluation of (pre)speech or babbling skills by a speech language pathologist is beneficial for all newly diagnosed FDS patients. | 8.3 (1) | 19 | V | - | - | - |
|  |  |  |  |  |  |  |
| **Hearing** |  |  |  |  |  |  |
| 144. Recommendations from the consensus statement on bone conduction devices and active middle ear implants in conductive and mixed hearing loss, are applicable to FDS patients | 7.6 (0) | 12 | V | N/A | Maier (2022) | ^80^ |
| 145. Prior to surgery for hearing, health care providers should provide information on the different hearing devices, their principles of action, (dis)advantages, longevity, upgrade options, and MRI-compatibility. | 8.8 (0) | 10 | V | - | Maier (2022) | ^49^ |
| 146. Audiologic screening soon after birth is necessary to allow for timely treatment of hearing loss in FDS patients. | 8.6 (0) | 11 | V | - | - | - |
| 147. In case of bilateral hearing loss in FDS patients, hearing amplification of at least one ear is necessary. | 8.7 (0) | 11 | V | - | - | - |
| 148. Bilateral hearing amplification can achieve better sound localisation and lateralisation compared to unilateral hearing amplification in FDS patients with bilateral conductive hearing loss | 8.3 (0) | 12 | V | - | - | - |
| 149. The maximum power output (MPO) in dB is helpful to compare hearing devices and assess the device for an FDS patient's hearing function. | 7.8 (0) | 8 | V | - | Maier (2022) | ^49^ |
| 150. FDS patients with conductive or mixed hearing loss who are too young for an implant, can benefit from early hearing amplification with non-implantable hearing devices | 8.3 (1) | 12 | III | Average | Verhagen (2008);  Urik (2019) | ^81,82^ |
| 151. For FDS patients with malformations of the middle or outer ears resulting in conductive or mixed hearing loss, bone-anchored hearing aids or bone-conduction hearing aids are appropriate options. | 7.7 (1) | 12 | IV | Average | Herrmann (2005);  Pron (1993) | ^45,48^ |
| 152. In FDS patients, implantable hearing devices are helpful if reconstructive middle ear surgery is not feasible, if long-term outcomes of usual surgical procedures expected to be poor, if fitting of conventional hearing devices is contraindicated (behind-the-ear devices, non-surgical bone conductors), or if fitting of conventional hearing devices is expected to give unsatisfactory outcomes. | 8.1 (1) | 12 | IV | Average | Mylanus (1998); Nadaraja (2013);  De Wolf (2010) | ^50-52^ |
| 153. In FDS patients, transcutaneous and percutaneous bone conduction implants, and active middle ear implants can be considered as implantable devices for treatment of hearing loss | 8.2 (1) | 9 | V | - | Maier (2022) | ^49^ |
| 155. Bone-anchored hearing aid placement can improve the general well-being and quality of life of FDS patients with hearing loss. | 7.8 (1) | 12 | IV | High | Marsella (2011) | ^83^ |
| 156. Bone-anchored hearing aids are superior to conventional bone conduction hearing aids in improving hearing (in dB) in FDS patients with hearing loss. | 8.0 (1) | 10 | IV | Average | McDermott (2009) | ^84^ |
| 157. CT scanning of the temporal bones for middle ear evaluation can be used to determine candidacy for middle ear surgery. | 8.5 (0) | 8 | IV | Average | Herrmann (2005);  Jahrdoerfer (1989); Marres (1995) | ^45,46,85^ |
| 158. Currently, there is no conclusive evidence of the benefit of surgical correction of external auditory canal stenoses or ossicular fixations to treat conductive hearing loss in FDS patients. | 7.6 (1) | 8 | IV | Average | Herrmann (2005) | ^45^ |
| 159. Bone-conduction or bone-anchored hearing aids are preferred over reconstructive middle ear and/or aural canal surgery for hearing rehabilitation in FDS patients with congenital aural atresia. | 8.4 (0) | 11 | IV | Average | Marres (1995) | ^46^ |
| 160. Regular otologic and audiologic screening is beneficial for FDS patients to monitor hearing, irrespective of the severity of ear malformations | 8.4 (0) | 9 | IV | Average | Herrmann (2005); Marres (1995);  Plomp (2013) | ^45-47^ |
| 214. Audiologic follow-up examinations are preferable at least every three years in FDS patients to monitor hearing. | 7.6 (1) | 9 | V | - | - | - |
| 161. Audiological examination before hearing treatment and four weeks after the acclimatization period is preferable to evaluate changes in hearing in FDS patients | 8.0 (1) | 9 | V | - | - | - |
| 162. FDS patients with minor ear anomalies may still experience severe hearing loss and can benefit from hearing aids as early as possible. | 8.7 (0) | 10 | IV | Average | Marres (1995) | ^46^ |
| 215. Air conducting hearing devices are an appropriate first choice in FDS patients if acceptable hearing and speech intelligibility can be achieved and their ear anatomy allows for wearing them. | 8.5 (1) | 8 | V | - | - | - |
| 216. The choice of hearing device should be individualized for each patient, considering the type(s) of hearing loss (conductive, sensorineural, mixed), physical anatomy, and the preferences of parents and patients. | 8.8 (0) | 10 | V | - | - | - |
| 217. There is no evidence available whether hearing loss is progressive in some FDS patients. | 8.0 (1) | 6 | V | - | - | - |
| 220. In case of hearing loss in FDS patients, routine monitoring of hearing is preferably performed throughout hearing rehabilitation | 8.8 (0) | 10 | V | - | - | - |
|  |  |  |  |  |  |  |
| **Dental treatment and Orthodontics** |  |  |  |  |  |  |
| 164. FDS patients benefit from biannual (=every 6 months) dental evaluation to prevent oral diseases. | 7.9 (0) | 15 | V | - | - | - |
| 165. FDS patients benefit from biannual dental evaluation to monitor growth and development and allow for timely diagnosis and treatment of dental problems. | 7.3 (1) | 15 | V | - | - | - |
| 167. FDS patients may require lifelong multidisciplinary health services related to their oral condition, regardless of surgical interventions, to ensure optimal oral health. | 8.5 (0) | 21 | III | High | Åsten (2013) | ^53^ |
| 168. FDS patients may benefit from at least one dental screening to assess for dental anomalies | 8.7 (0) | 19 | IV | Average | da Silva Dalben (2006) | ^55^ |
| 169. The monitoring and documentation of dental problems in FDS patients can be carried out in a manner equivalent to that used for patients with other craniofacial anomalies. | 8.4 (1) | 17 | V | - | - | - |
| 170. Salivary gland dysfunction (i.e., low secretion rates) cannot reliably be detected by ultrasound imaging in FDS patients. | 7.1 (0) | 8 | III | Average | Osterhus (2012) | ^54^ |
|  |  |  |  |  |  |  |
| **Growth, Feeding, and Swallowing** |  |  |  |  |  |  |
| 171. Currently, evidence regarding differences in nutritional status, such as BMI, between adults with and without FDS is lacking | 7.6 (0) | 15 | III | High | Medeiros (2022) | ^86^ |
| 173. (Multidisciplinary) treatment strategies for feeding and swallowing problems in FDS patients should be further developed | 8.2 (1) | 22 | V | - | - | - |
| 174. FDS patients may benefit from routine screening for feeding problems (dysphagia) to allow for timely diagnosis and treatment. | 7.8 (1) | 18 | V | - | - | - |
| 175. In case of (suspected) feeding and/or swallowing problems, evaluation of feeding patterns by a preverbal speech therapist is preferable | 8.6 (0) | 19 | V | - | - | - |
| 177. In FDS patients between the ages of 1-6 years, annual measurements of growth (length, weight) are preferable. | 7.7 (1) | 20 | V | - | - | - |
| 178. In case of insufficient growth of FDS patients, obstructive sleep apnea (OSA) and feeding problems should be considered as possible causes. | 8.5 (1) | 21 | V | - | - | - |
|  |  |  |  |  |  |  |
| **Extracranial Anomalies** |  |  |  |  |  |  |
| 179. FDS patients with limb anomalies are preferably referred to a center with expertise as soon as possible to inform and guide the parents | 9.0 (0) | 9 | V | - | - | - |
| 182. In newly diagnosed FDS patients, a comprehensive screening for extracranial anomalies is preferable, particularly vertebral and cardiac, as well as limb anomalies in Nager and Miller syndrome. | 9.0 (0) | 7 | III | High | Beaumont (2021); Panamonta (2018);  Pun (2012) | ^57-59^ |
| 183. Screening for (multiple) vertebral anomalies in FDS patients should ideally cover at least spina bifida occulta, dysmorphic C1, scoliosis, reduced C2/C3 space, dysmorphic spinous process(es), and pectus excavatum or carinatum. | 8.6 (0) | 7 | III | High | Beaumont (2021);  Pun (2012) | ^57,59^ |
| 184. Screening for cardiac anomalies in FDS patients should ideally cover at least atrial septal defect, patent ductus arteriosus, and ventricular septal defect. | 7.3 (1) | 7 | III | High | Beaumont (2021); Panamonta (2018) | ^57,58^ |
| 186. Parents (and FDS patients once they are able to understand) should be informed about the possible cardiac and vertebral anomalies to allow for timely detection | 8.7 (0) | 9 | V | - | - | - |
|  |  |  |  |  |  |  |
| **Psychology and Cognition** |  |  |  |  |  |  |
| 187. Starting with the parents and later including the patient, psychological family assessment is helpful to evaluate motivations, expectations, and the family's planned involvement regarding facial reconstructive surgery, as well as coping style and resilience. | 8.4 (0) | 18 | IV | Average | Arndt (1987) | ^60^ |
| 188. The combination of surgery and psychological support may enhance long-term psychological and social functioning of FDS patients | 8.7 (0) | 18 | IV | High | Versnel (2012); van den Elzen (2012); Arndt (1987) | ^60-63^ |
| 190. If questions regarding the cognitive development of a child with FDS arise, cognitive assessment should preferably be offered without delay by a qualified (neuro)psychologist. | 8.0 (1) | 17 | V | - | - | - |
| 191. If the FDS patient and/or their parent(s) suffer from anxiety at any point during treatment, assessment for psychological needs is preferable to allow for timely management | 8.6 (0) | 21 | V | - | - | - |
| 192. If life threatening situations occur for the FDS patient (e.g., risk of suffocation), psychological trauma in patient and parent(s) should preferably be screened for. | 7.7 (0) | 16 | V | - | - | - |
| 193. Parents of FDS patients should preferably have the opportunity to receive pedagogical and/or psychological support by a psychologist and/or social worker. | 8.2 (0) | 19 | V | - | - | - |
| 195. Play therapy (e.g. surgery on a teddy bear) can be helpful to prepare a young pediatric FDS patient psychologically for (surgical) procedures. | 7.7 (1) | 16 | V | - | - | - |
|  |  |  |  |  |  |  |
| *In case of multiple sources, highest level of evidence and quality were stated. |  |  |  |  |  |  |
