# Supplementary material for: Optimal Diagnostic and Treatment Practices for Facial Dysostosis Syndromes: A Clinical Consensus Statement Among European Experts
Source: J Craniofac Surg. 2024 May 27;35(5):1315–24. doi: 10.1097/SCS.0000000000010280 (PMC11198962; doi:10.1097/SCS.0000000000010280)
Supplement: Supplementary file 2 [file scs-35-1315-s002.docx]

Supplemental Digital File 1 – Search Strings of the Systematic Literature Search

| **Database searched** | **Platform** | **Years of coverage** | **Records** | **Records after duplicates removed** |
| --- | --- | --- | --- | --- |
| Medline ALL | Ovid | 1946 - Present | 2319 | 2316 |
| Embase | Embase.com | 1971 - Present | 2619 | 710 |
| Web of Science Core Collection* | Web of Knowledge | 1975 - Present | 1308 | 317 |
| Cochrane Central Register of Controlled Trials | Wiley | 1992 - Present | 4 | 1 |
| **Total** | | | **6250** | **3344** |

*Science Citation Index Expanded (1975-present) ; Social Sciences Citation Index (1975-present) ; Arts & Humanities Citation Index (1975-present) ; Conference Proceedings Citation Index- Science (1990-present) ; Conference Proceedings Citation Index- Social Science & Humanities (1990-present) ; Emerging Sources Citation Index (2005-present)

No other database limits were used than those specified in the search strategies

**Medline 2319**

(Mandibulofacial Dysostosis / OR (((mandibulofacial* OR facial* OR Nager OR acrofacial* OR miller) ADJ3 (dysostos*)) OR ((Postaxial* OR Post-axial*) ADJ3 (acrofacial* OR acro-facial) ADJ3 dysostos*) OR (Treacher ADJ3 Collins) OR Franceschetti*).ab,ti,kw.) NOT (news OR congres* OR abstract* OR book* OR chapter* OR dissertation abstract*).pt. NOT (exp animals/ NOT humans/)

**Embase 2619**

('mandibulofacial dysostosis'/de OR 'Nager acrofacial dysostosis'/de OR (((mandibulofacial* OR facial* OR Nager OR acrofacial* OR miller) NEAR/3 (dysostos*)) OR ((Postaxial* OR Post-axial*) NEAR/3 (acrofacial* OR acro-facial) NEAR/3 dysostos*) OR (Treacher NEAR/3 Collins) OR Franceschetti*):Ab,ti) NOT ([conference abstract]/lim) NOT ([animals]/lim NOT [humans]/lim)

**Web of science 1308**

TS=(((((mandibulofacial* OR facial* OR Nager OR acrofacial* OR miller) NEAR/2 (dysostos*)) OR ((Postaxial* OR Post-axial*) NEAR/2 (acrofacial* OR acro-facial) NEAR/2 dysostos*) OR (Treacher NEAR/2 Collins) OR Franceschetti*))) NOT DT=(Meeting Abstract OR Meeting Summary) AND LA=(English)

**Cochrane 4**

((((mandibulofacial* OR facial* OR Nager OR acrofacial* OR miller) NEAR/3 (dysostos*)) OR ((Postaxial* OR Post-axial*) NEAR/3 (acrofacial* OR acro-facial) NEAR/3 dysostos*) OR (Treacher NEAR/3 Collins) OR Franceschetti*):Ab,ti)
