# Supplementary material for: Optimal Diagnostic and Treatment Practices for Facial Dysostosis Syndromes: A Clinical Consensus Statement Among European Experts
Source: J Craniofac Surg. 2024 May 27;35(5):1315–24. doi: 10.1097/SCS.0000000000010280 (PMC11198962; doi:10.1097/SCS.0000000000010280)
Supplement: Supplementary file 4 [file scs-35-1315-s004.docx]

**Supplemental Digital File 4 – Available Hearing Devices**

**Percutaneous bone conduction devices**

| Baha® Connect, Cochlear BAS, Gothenburg, Sweden |
| --- |
| Ponto system, Oticon Medical AB, Askim, Sweden |

**Transcutaneous passive bone conduction devices**

| Baha® Attract, Cochlear BAS, Gothenburg, Sweden |
| --- |
| Sophono, Medtronic, Jacksonville, FL |

**Transcutaneous active bone conduction devices**

| Bonebridge, Med-El, Innsbruck, Austria |
| --- |
| Osia, Cochlear BAS Gothenburg, Sweden |
| Sentio, Oticon Medical AB, Askim, Sweden |

**Active middle ear implant**

| Vibrant Soundbridge device or VSB, Med-El, Innsbruck, Austria |
| --- |
